# Atypical Asymmetry for Processing Human and Robot Faces in Autism Revealed by fNIRS

**DOI:** 10.1371/journal.pone.0158804

**Published:** 2016-07-07

**Authors:** Corinne E. Jung, Lars Strother, David J. Feil-Seifer, Jeffrey J. Hutsler

**Affiliations:** 1 Department of Psychology, Program in Neuroscience, University of Nevada, Reno, Reno, Nevada, United States of America; 2 Department of Computer Science and Engineering, University of Nevada, Reno, Reno, Nevada, United States of America; University of Tuebingen Medical School, GERMANY

## Abstract

Deficits in the visual processing of faces in autism spectrum disorder (ASD) individuals may be due to atypical brain organization and function. Studies assessing asymmetric brain function in ASD individuals have suggested that facial processing, which is known to be lateralized in neurotypical (NT) individuals, may be less lateralized in ASD. Here we used functional near-infrared spectroscopy (fNIRS) to first test this theory by comparing patterns of lateralized brain activity in homologous temporal-occipital facial processing regions during observation of faces in an ASD group and an NT group. As expected, the ASD participants showed reduced right hemisphere asymmetry for human faces, compared to the NT participants. Based on recent behavioral reports suggesting that robots can facilitate increased verbal interaction over human counterparts in ASD, we also measured responses to faces of robots to determine if these patterns of activation were lateralized in each group. In this exploratory test, both groups showed similar asymmetry patterns for the robot faces. Our findings confirm existing literature suggesting reduced asymmetry for human faces in ASD and provide a preliminary foundation for future testing of how the use of categorically different social stimuli in the clinical setting may be beneficial in this population.

## Introduction

Distinct cognitive and behavioral deficits observed in ASD may be attributed to altered patterns of functional hemispheric asymmetry[1]. Less asymmetry is represented by reduced hemisphere dominance and more bilateral recruitment of homologous regions of the cortex [2]. This could indicate inefficient cognitive functioning that may lead to observable deficits in domains like language and facial processing, which are typically lateralized in NTs [3]. Atypical patterns of asymmetry have been identified in ASD, but most of these studies have only investigated asymmetry for language processing, which is generally left lateralized in NT individuals [4]. Imaging studies have shown mixed results, including reduced asymmetry [4,5], reversed patterns of asymmetry [6], and typical asymmetry [7]. However, the theory of reduced asymmetry for hemisphere dominant tasks, such as language and facial processing, most consistently supports and explains the deficits characteristically observed in ASD.

Hemisphere dominance has been theorized to promote efficiency by avoiding duplication of functions in both hemispheres and allowing different categories of information to be processed in parallel [8]. Reduced asymmetry suggests that both hemispheres participate more equally in processing these tasks. As such, performance may suffer; both hemispheres are weakly activated, neither being dominant for the task[9]. In ASD, research has shown volume reductions in regions of the corpus callosum, the structure that connects the hemispheres and impacts the development of lateralization[10]. Alterations to this structure could impact functional asymmetry by interfering with the isolation required for each hemisphere to develop dominance in specific domains[11].

Imaging data has shown greater bilateral activation in ASD, compared to NT groups that demonstrate more left hemisphere activation, for receptive language tasks [5], as well as reduced leftward asymmetry in prefrontal regions for language production tasks [4]. Recent evidence suggests that the development of facial processing and language are jointly impacted [12–17],suggesting that asymmetry for both of these domains may complement each other.

Facial processing deficits in ASD appear in a range of behavioral studies from gender discrimination, recognition [18], emotion identification [19], and memory for faces [20]. Functional imaging has shown normal early visual processing in ASD[21], suggesting that facial processing deficits likely arise from problems with higher-level cognitive functioning, rather than general visual processing. The fusiform face area (FFA), a region in the fusiform gyrus, and the occipital face area (OFA), a region in the occipital cortex, are specific areas that are engaged during facial processing tasks and more active in the right hemisphere in NTs[22]. The results of functional magnetic resonance imaging (fMRI) studies investigating these regions in ASD are not consistent. However, much research suggests that individuals with ASD do not exhibit the same right hemisphere asymmetry as NTs during facial processing, such as having weaker activation in the FFA[23,24] and OFA [25]. Due to the widespread temporal-occipital activity around the FFA and OFA in response to facial stimuli in NTs [26], this broader facial network should be further examined in ASD to more generally identify reduced right hemisphere asymmetry for facial processing.

Although facial processing deficits are well established in ASD[27,28],research has just begun to explore whether these deficits are limited to human faces. Although individuals with high functioning ASD show reduced activation of the facial processing network in response to human faces, they elicit typical activation in this network in response to animal faces[25]. Additionally, research shows that children with ASD have increased verbal utterances towards robots than towards people in the clinical setting [29]. This is further supported by increased speech directed at robots compared to adults [30], suggesting that differential processing for non-human faces can potentially be used to improve socialization in ASD. These findings can be bolstered by assessing indices of how the brain responds to robot stimuli in ASD, and how these patterns may be similar to, or different from, those of NTs.

The novel studies examining interaction between individuals with ASD and interactive robots did not incorporate an NT group for comparison, but, because functional imaging research in NTs has shown comparable FFA activity for cat, cartoon, and human faces [31], we predict that they would also show similar patterns of functional activation for human and robot faces. In contrast, if individuals with ASD do not treat all faces similarly, we should be able to objectively demonstrate this through a comparison of asymmetry for human faces and robot faces in this population. Therefore, assessing lateralization patterns for human versus robot faces in both groups may contribute to an explanation for the increased interactions for robot over human counterparts in ASD.

FNIRS is a useful method for measuring and comparing cortical activation patterns in children and special populations because it allows participants to sit openly in a chair without being confined within a scanner or exposed to a magnetic field and loud noises. This noninvasive imaging technique has been used in individuals with ASD to assess interhemispheric connectivity during the resting state [32], and in prefrontal areas [33]and has suggested reduced connectivity in this population. In this preliminary experiment, we used fNIRS to compare the degree of asymmetry in the temporal-occipital regions in an ASD and an NT group for tasks that required processing of both human and robot faces. Because individuals with ASD show deficits in basic facial processing tasks, our first objective was to test the hypothesis that the ASD group in our sample would show reduced lateralization for human face stimuli relative to the NT group. Second, because individuals with ASD show improved interactions with robot faces, we wished to see whether these stimuli would show lateralization in the ASD group. Comparing patterns of activation for the robot faces in both groups could provide preliminary support for the successful use of robots in a clinical setting and encourage more effective methods of stimulating interaction.

## Methods

### Participants

Participants in this study included 8 males with a diagnosis of ASD and 12 NT males. All participants were between the ages of 7 and 36 (NT: M = 14.5, SD = 10.76; ASD: M = 15.6, SD = 9.55). To account for the heterogeneous nature of our sample, we ensured that each participant with ASD that was 22 years of age and younger was age matched with at least one NT participant within one year of age. For the one adult participant with ASD who was over 30 years old, we simply ensured we matched him with an NT participant within 10 years of age, since the significant developmental changes that occur early in life do not occur in adulthood. All procedures were conducted in accordance with the University of Nevada, Reno Institutional Review Board, following its approval of this study. Participants, or parents of participants under 18 years of age, signed written consent forms, and all participants provided verbal and non-verbal assent prior to and throughout the testing. Individuals in the ASD group had previously been diagnosed with ASD by a licensed clinical psychologist or doctor, not associated with this research. All participants in the ASD group had also been previously assessed with the Autism Diagnostic Observation Schedule (ADOS) by a clinical psychologist or speech pathologist qualified to administer this assessment. Individuals in the NT group had no previous diagnosis of ASD or a history of a medical disorder known to cause characteristics associated with ASD.

All participants were further assessed using the Wechsler Abbreviated Scale of Intelligence (WASI) to obtain a Full-2 IQ [34] (NT: M = 112.2, SD = 12.45; ASD: M = 95.9, SD = 14.95), the Edinburgh Handedness Inventory [35] to determine a laterality quotient (LQ) (NT: M = 82, SD = 15.21; ASD: M = 61.1, SD = 39.45), and the Gilliam Autism Rating Scale, Second Edition GARS-2[36]to obtain an Autism Index for the ASD group only (M = 89.9, SD = 22.75). Participants or parents of the participants under 18 years of age, filled out information for the Edinburgh Handedness Inventory. They indicated the individual’s hand dominance for 10 different everyday activities so that we could obtain an LQ for each participant. For the GARS-2, parents or caregivers provided current ratings of how often they observed the individual exhibit certain characteristics in the following categories: stereotyped behaviors, communication, and social interaction (Table 1).

**Table 1:** Participant characteristics.

Participant Classification	Age	WASI Full-2 IQ	LQ (-100-100)	GARS-2
NT 1	9	112	67	N/A
NT 2	10	117	100	N/A
NT 3	12	116	100	N/A
NT 4	7	132	85	N/A
NT 5	9	131	67	N/A
NT 6	14	99	88	N/A
NT 7	7	109	90	N/A
NT 8	13	88	79	N/A
NT 9	7	113	100	N/A
NT 10	18	119	88	N/A
NT 11	23	105	60	N/A
NT 12	45	105	60	N/A
ASD 1	10	81	100	74
ASD 2	11	124	38	128
ASD 3	9	104	71	59
ASD 4	18	91	-11	89
ASD 5	11	83	100	89
ASD 6	36	104	50	109
ASD 7	8	81	100	81
ASD 8	22	99	41	N/A

### Stimuli and Procedure

**Fig 1:**
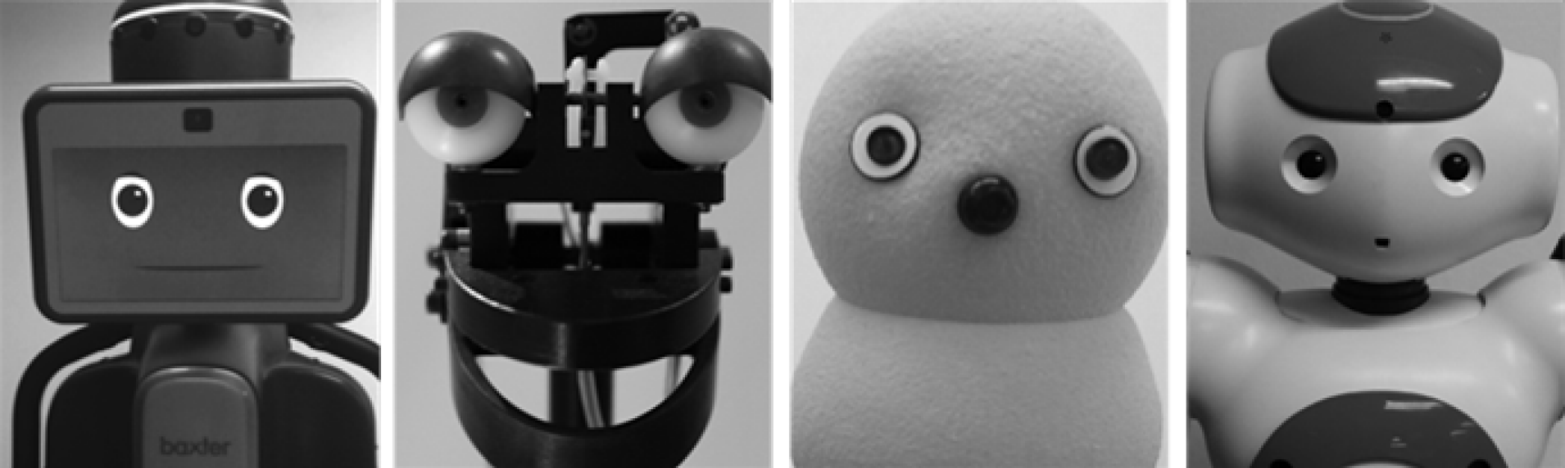
Examples of stimuli. The robot face task consisted of robots with distinct head shapes and key facial feature landmarks. Reprinted from Elaine Short under a CC BY license, with permission from Elaine Short, original copyright 2014.

During the experiment, each participant was seated 57 cm away from a 17 inch Dell laptop, and all stimuli were presented using E-prime software [37]. To keep participants engaged in the stimuli, they were asked to do a 1-back task, in which they were instructed to press one key when the current stimulus repeated from the one prior and another key if it was different. The 1-back task was used to keep participants actively involved with the stimuli, rather than passively looking at the screen[38]. Face stimuli in the human task were acquired from a stimuli set used in previous research [39] and included pictures of 5 males and 5 females, without glasses or jewelry, showing neutral expressions. Facial stimuli with neutral expressions were chosen for two important reasons. First, they are more comparable to the emotionally neutral robot faces. More importantly, research suggests that individuals with ASD identify and process emotion differently than NTs [19,40,41]. Thus, emotionally neutral stimuli were used to eliminate the potential fora group difference in emotion identification to be a confounding variable. Face stimuli in the robot task were acquired from the UNR Robotics Research Lab, the Yale Social Robotics Lab, and the USC Interaction Lab and included pictures taken of 10 robots used in laboratory, research, or clinical settings that all had distinguishable heads and facial feature landmarks (Fig 1). All stimuli were grayscale, subtending 8° visual angle horizontally and 10° vertically, similar to the size of a real face viewed from about 100 cm away [42].

There were 10 blocks of human faces and 10 blocks of robot faces. Each block consisted of 15 stimuli presented successively. The sequences of presentations per trial that participants viewed for the 1-back task were created as follows: From the set of 10 possible images for the stimuli type, 15 image presentations, and whether or not the image would repeat from the previous, were determined randomly. More specifically, each image was labeled 1–10 and, for the first presentation, an image from 1–10 was randomly generated. For the following image, whether it would repeat was determined randomly. If it was a repeat, the same image would be used for the second position, but if it was not a repeat, an image would be randomly generated again. The process repeated for the 15 presentations per trial. Finally, this entire process was repeated for 10 total blocks. This procedure was performed identically for the human and the robot trials. Each participant received the same sequence of stimuli and trials. Each stimulus remained on the monitor for 1000 ms, with fixed 500 ms interstimulus intervals that were not dependent on key responses. After the last stimulus of each block, there was an 8 second rest period. This resting time gave the hemodynamic response adequate time to return back to baseline [43–45], while still keeping the total experiment time comfortable for the patient population. The order in which the blocks were presented was randomized. The entire experimental session lasted about 12 minutes. Prior to beginning fNIRS recording, each participant ran through 4 randomized blocks (60 stimuli) of the behavioral task once for practice (for results of behavioral task, see S1 Fig).

### FNIRS set up

**Fig 2:**
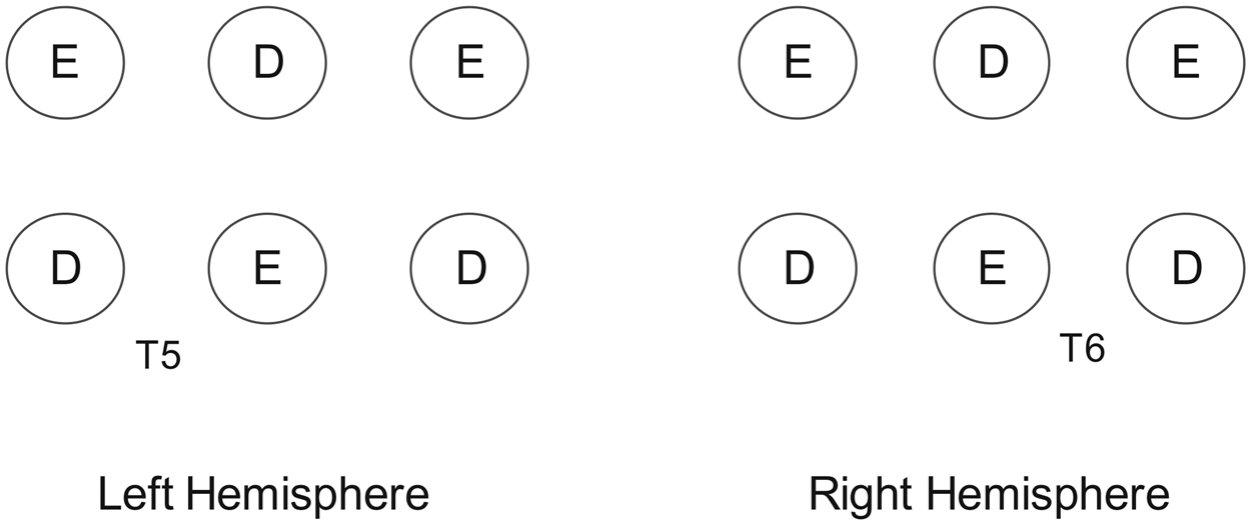
Array depicting placement of emitter (E) and detector (D) probes over back of head.

Oxygenated (HbO) and deoxygenated (HbR) hemoglobin levels were measured with a continuous wave fNIRS system (TechEn CW6 fNIRS System, Milford, MA), measuring two wavelengths (690 nm and 830 nm) that were sampled at 50 Hz (20 ms). Each participant’s head was measured using the international 10–20 system as a reference for probe placement [46]. Specifically, channels in the left hemisphere were placed such that one emitter-detector pair was directly over T5, with six other channels placed around it, and channels in the right hemisphere were placed symmetrically, with one directly over T6, for a total of 14 channels (Fig 2). Bilateral temporal areas include T5 and T6, and asymmetry for faces has previously been detected in these regions with fNIRS[47]. The path of light from the emitter to the detector travels in a shallow banana shaped path that only penetrates approximately half the distance between the optodes[48], and it is therefore recommended that the distance between each emitter and detector be at least 2.5 cm. Thus, we set optical channels at 2.6 cm in distance in a 2x3 lattice attached to a custom-made headband to ensure that the configuration was constant within each hemisphere for all participants. Prior to beginning the experiment, the channels were screened for clear respiratory patterns at both wavelengths. If the signal was not clear enough to see the respiratory pattern and cardiac pulsation, we adjusted the setup on the participant’s head. Any channels that remained noisy were noted for later exclusion from data analysis.

### Analysis

Oxygenation concentration changes from baseline from raw signals were obtained using the modified Beer-Lambert approach [49] and analyzed with the HomER2 software package [50]. Raw fNIRS data were pre-processed with a low pass filter of 0.5 Hz to eliminate respiratory noise, heart pulsation, and high frequency noise in the signal. We visually inspected the waveforms for motion artifacts, but none were found, and we did not apply any further motion artifact removal techniques. As HbO has previously been shown to correlate best with blood flow compared to HbR and total hemoglobin values [51], we focused on HbO signals for analysis (for sample raw data, see S2 Fig).

Data was obtained for each 20 ms of recording, which we averaged to get values for each second. To compare relative HbO values across channels and participants, we normalized the raw scores into Z-scores, as this allows data to be averaged regardless of unit[52,53]. We based these scores on the baseline rest period starting from 5 seconds prior to the task period. Thus, each z-score represents the change of the hemodynamic response during the presentation of the faces from baseline. Z-scores were calculated for every channel at each second as follows: (*x*_*task*_−*m*_*baseline*_)/*s* [47]. *x*_*task*_ represents the raw data at each second during the task period, *m*_*baseline*_ represents the mean of the raw data during the last 5 seconds of the baseline period, and *s* represents the standard deviation of the raw data during baseline.

For each hemisphere, we calculated the average HbO levels across channels at each second with these normalized scores [54]excluding channels that did not meet our predetermined criteria for an acceptable signal, as well as corresponding channels in the opposite hemisphere. This provided average values at each time point for the left and right hemisphere. To account for the rise of the hemodynamic response from resting levels [55,56], we then took the peak HbO level from those averages in the 3 to 10 second time frame after the face stimuli onset. Finally, there were data values representing peak activation for the left and right hemispheres for each participant in both the task for human faces and the task for robot faces. To test our first hypothesis that the ASD group would show less asymmetry than the NT group for human faces, we conducted a 2x2 repeated-measures ANOVA for the human faces condition, with a between-subjects factor of diagnosis (ASD and NT) and a within-subjects factor of hemisphere (left and right). To test our second question of whether the ASD group would show more comparable asymmetry to the NT group, we conducted a 2x2 repeated-measures ANOVA for the robot faces condition, also with a between-subjects factor of diagnosis and a within-subjects factor of hemisphere. We conducted these as two separate analyses because the first analysis served to use our sample to support the preexisting literature, while the second analysis served to test a novel question. The participants’ variables of age, IQ, and LQ were later included as covariates to assess their impact, if any, on the overall findings.

## Results

Analyses of the assessments showed that the groups did not significantly differ on age or LQ. There was a significant difference in IQ between groups, but further analysis showed that, of the vocabulary and matrix reasoning subcomponents that made up the IQ score, only vocabulary significantly differed between groups. Although groups are generally matched on IQ to control for general cognitive abilities, the diagnostic profile for ASD suggests that matching groups for performance on tests that utilize language skills may not be appropriate [57]. Language is characteristically aberrant in this population, and lower scores in vocabulary subcomponents of IQ tests are not unusual [57–61]. Therefore, we felt that it was only critical that the groups did not significantly differ on the matrix reasoning task, a measure of abilities that are generally not impaired in ASD [62].

**Fig 3:**
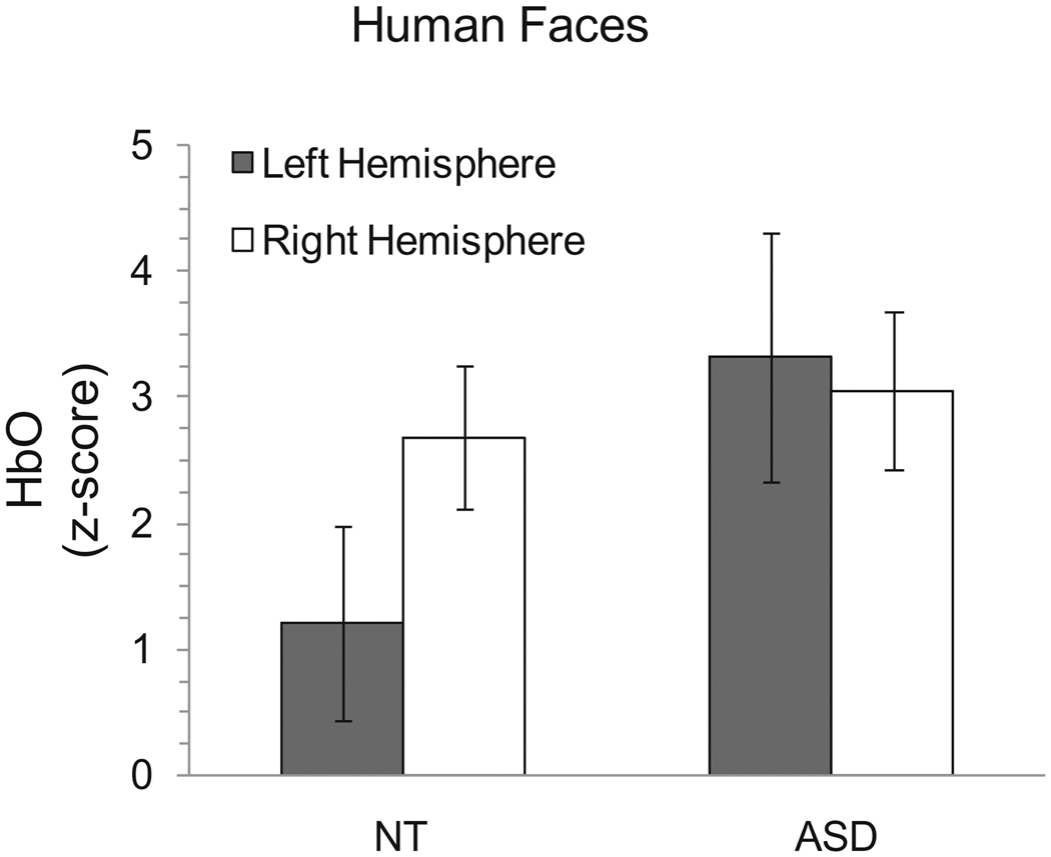
HbO z-scores for the human faces condition for both groups. The NT group shows greater right hemisphere lateralization than the ASD group. All error bars represent standard error of the mean.

After excluding all the channels due to poor signals, we found that the number of channels excluded in each group did not significantly differ (NT channel exclusions: M = 1.25, SE = .392; ASD channel exclusions: M = 1.5, SE = .378; *t*(18) = -.437, *p* = .389). The analysis of the fNIRS data for our investigation of the human faces showed that there was no main effect of hemisphere (*F*(1, 18) = 3.085, *p* = .096) or diagnosis (*F*(1, 18) = 1.476, *p* = .24). However, the interaction between hemisphere activity and diagnosis was significant(*F*(1, 18) = 6.12, *p* = .012) (Fig 3). Post-hoc pairwise comparisons, using Bonferroni correction for multiple comparisons, indicated that the NT group exhibited greater activation in the right hemisphere than the left hemisphere (*p* = .002), and there was no significant difference between the hemispheres for the ASD group (*p* = .649). Neither the left nor right hemisphere showed a significant difference between diagnoses (*p* = .105 and *p* = .712, respectively).

**Fig 4:**
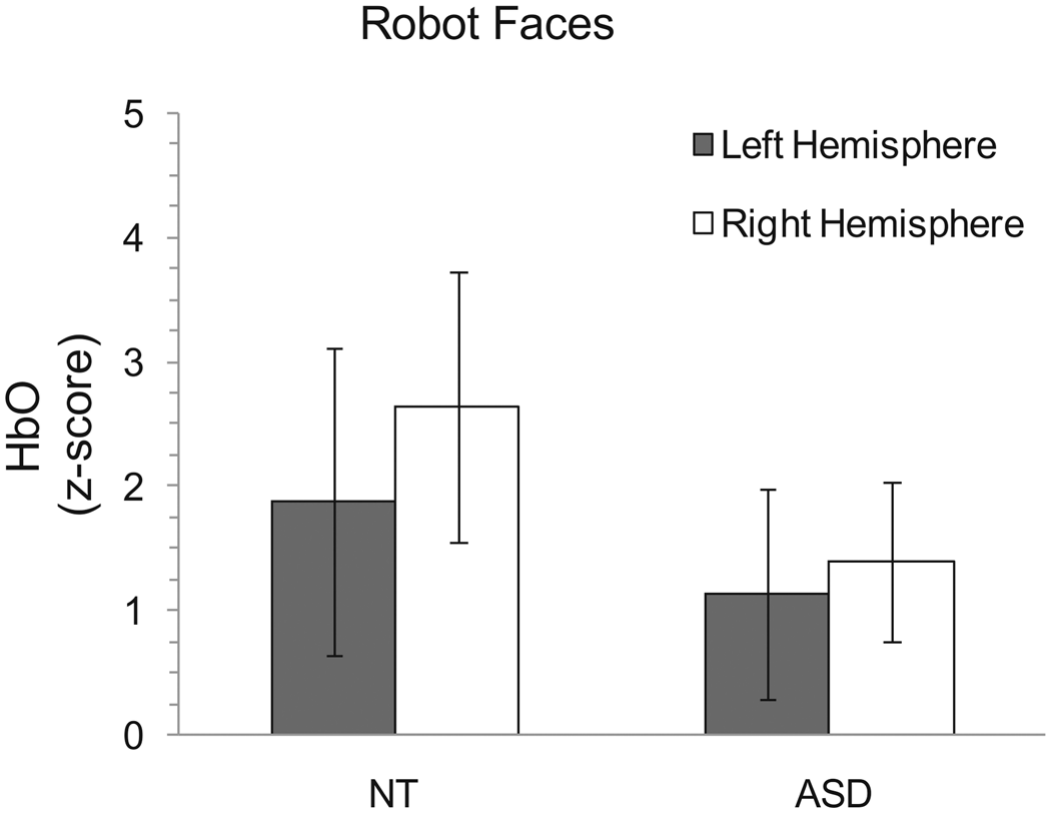
HbO z-scores for the robotic face condition for both groups. The groups do not differ in lateralized activation. All error bars represent standard error of the mean.

The analysis for our investigation of the robot faces showed that there was no main effect of hemisphere (*F*(1, 18) = 1.241, *p* = .28) or diagnosis (*F*(1, 18) = .444, *p* = .514). Additionally, there was no interaction between diagnosis and lateralization for robot faces (*F*(1, 18) = .298, *p* = .592) (Fig 4).

In each case, these results were upheld after adding age, IQ, and LQ as covariates. Finally, the GARS-2 scores for all ASD participants excluding one, who did not have a caretaker to provide scoring, did not predict left or right hemisphere activation for the ASD group in either the human faces condition (left: *r*(5) = .685, *p* = .089; right: *r*(5) = .346, *p* = .447) or the robot faces condition (left: *r*(5) = .116, *p* = .805; right: *r*(5) = .552, *p* = .229). Shapiro-Wilk tests confirmed that the residuals passed normality checks for both the human faces (SW (df = 20) = .971, *p* = .771) and the robot faces (SW (df = 20) = .924, *p* = .117).

## Discussion

This exploratory research used fNIRS to assess patterns of temporal-occipital brain activation in response to face stimuli in a group of NT individuals compared to a group with ASD. We tested two hypotheses regarding facial processing and asymmetry. First, we wanted to assess whether individuals with ASD elicit less lateralized activation in this facial processing region than NTs during facial processing tasks for typical human faces. Second, we wanted to determine whether lateralized activation patterns were similar between groups when viewing robot faces. As predicted, the NT group showed right hemisphere lateralization for the human faces, but the ASD group did not (Fig 3). Remarkably, however, brain activation patterns for the ASD group did not differ from the NT group for the robot faces (Fig 4). Taken together, these novel findings encourage future investigations for attributing characteristic behaviors in ASD to atypical functional asymmetry.

Previous reports suggesting functional alterations to asymmetry in ASD subjects in response to facial stimuli are currently limited. Here, we sought to bolster these studies and enhance the current understanding of abnormal asymmetry in this population. Reports of posterior corpus callosum structural abnormalities in ASD [63,64] are supported by these findings of reduced functional asymmetry for human faces, as deficits in this subsection could have inhibited development of asymmetry in face processing regions. Additionally, this finding complements the existing reports of reduced asymmetry for word processing in homologous regions because these processes generally arise in symmetrical regions of the cortex [65]. The second main finding that the ASD group's pattern of activation for the robot faces did not differ from the NTs, which suggests that the ASD group may be processing this category of faces similarly to NTs. Therefore, abnormal asymmetry is not globally observed in ASD. If the behavioral studies that showed individuals with ASD interact more with robots than humans [29]also tested an NT group, they may find that the interactions towards robots were comparable between groups, providing behavioral support for the data presented here.

Based on research that suggests NTs generalize across different categories of faces[31], this may not hold true in ASD. These individuals seem to process the robot faces similar to the way NTs do, with regards to lateralization. One possible explanation is that individuals with ASD exhibit more lateralized activity for objects and are processing robots more as objects, rather than as people. It has been suggested that they have greater social interest in object-like stimuli than human-like stimuli and that this interest in objects increases as they get older, so that they have even greater attraction to objects than NT children [66]. Additionally, research has supported typical, or even superior, object processing for individuals with ASD, compared to NTs [67,68]. For example, the N290, an event related potential (ERP) component that has shown face sensitivity, has faster responses to faces than objects in NTs, but faster responses to objects than faces in individuals with ASD [69].

Because we only assessed asymmetry for human and robot faces, it is difficult to reach the conclusion that the ASD participants were processing the robot faces like objects. A future study should incorporate an additional assessment of asymmetry in both groups for object stimuli that are complex enough to also be processed holistically or by their features, such as houses. This would help provide more insight as to the reason why individuals with ASD process robot faces differently than human faces. Additionally, longer rest periods can be incorporated. Although literature suggests that 8 seconds is adequate for the hemodynamic signal to return to baseline [43–45], other research suggests that a longer period of 12–15 seconds is ideal for allowing the signal to fully return to baseline [70]. Another limitation is the relatively small sample size. This exploratory study was intended to shed light on recent observations in the clinical setting with a more objective and theoretical framework. However, these findings warrant and encourage additional studies with larger sample sizes to further pursue these questions and validate the main findings and covariate analyses. The heterogeneous age sample we used could have influenced the results, as age has been shown to impact the degree of lateralization in NTs [2,71]. However, it is not clear exactly when lateralization progresses, or whether these changes are strictly due to maturational change [72]. Thus, we decided to incorporate a wider age range to help reduce any biases. Additionally, we believed that including both children and adults better represented the full breadth of developmental and learned changes that occur in both populations.

The results from this experiment lend support to the hypothesis that individuals with ASD show reduced functional lateralization while processing human faces. Similar patterns of asymmetrical activation in both groups when viewing robot faces suggests that facial processing may not be abnormal in ASD for all categories of face stimuli. This research can potentially be used to explain and determine cognitive performance in individuals with ASD, based on the patterns of abnormal functional asymmetry. Future studies can build on these results by assessing asymmetry for other lateralized domains that are associated with functions impacted in ASD.

## Supporting Information

S1 Fig Behavioral data for the 1-back task. No significant main effect of condition (*F*(1, 18) = 1.088, *p* = .311) or diagnosis (*F*(1, 18) = 2.335, *p* = .144). No significant interaction (*F*(1, 18) = .743, *p* = .4). All error bars represent standard error of the mean.
(TIF)

S2 Fig Example of raw time course data. FNIRS data from sample NT participant that includes HbO and HbR time course from each channel.
(TIF)

S1 File CC BY. (DOCX)

S2 File Data file. (XLSX)
