# Supplementary figures and images for: Atypical Asymmetry for Processing Human and Robot Faces in Autism Revealed by fNIRS
Source: PLoS One. 2016 Jul 7;11(7):e0158804. doi: 10.1371/journal.pone.0158804 (PMC4936708; doi:10.1371/journal.pone.0158804)

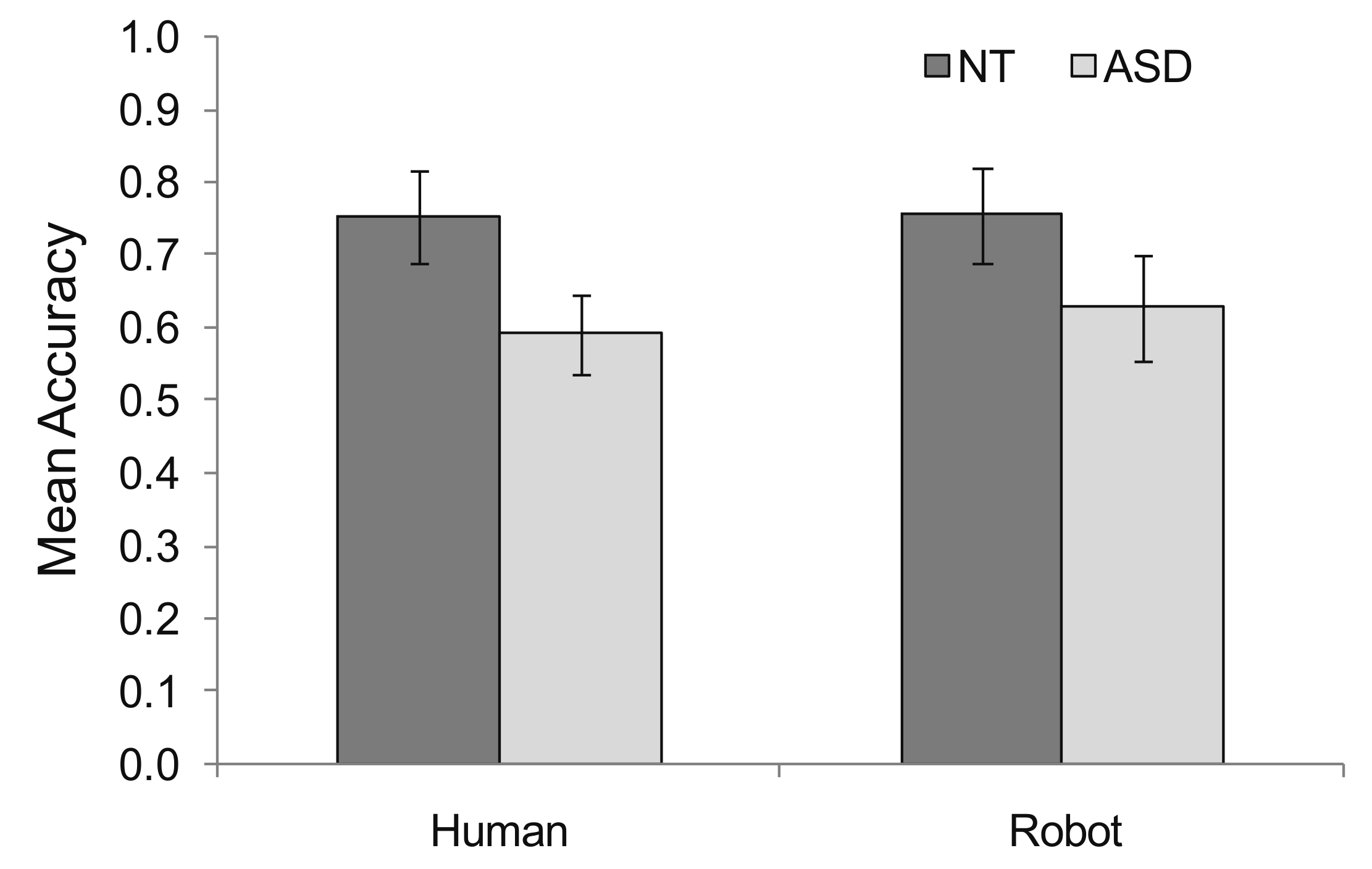

Supplement: S1 Fig — No significant main effect of condition (F(1, 18) = 1.088, p = .311) or diagnosis (F(1, 18) = 2.335, p = .144). No significant interaction (F(1, 18) = .743, p = .4). All error bars represent standard error of the mean. (TIF) [file pone.0158804.s001.tif]

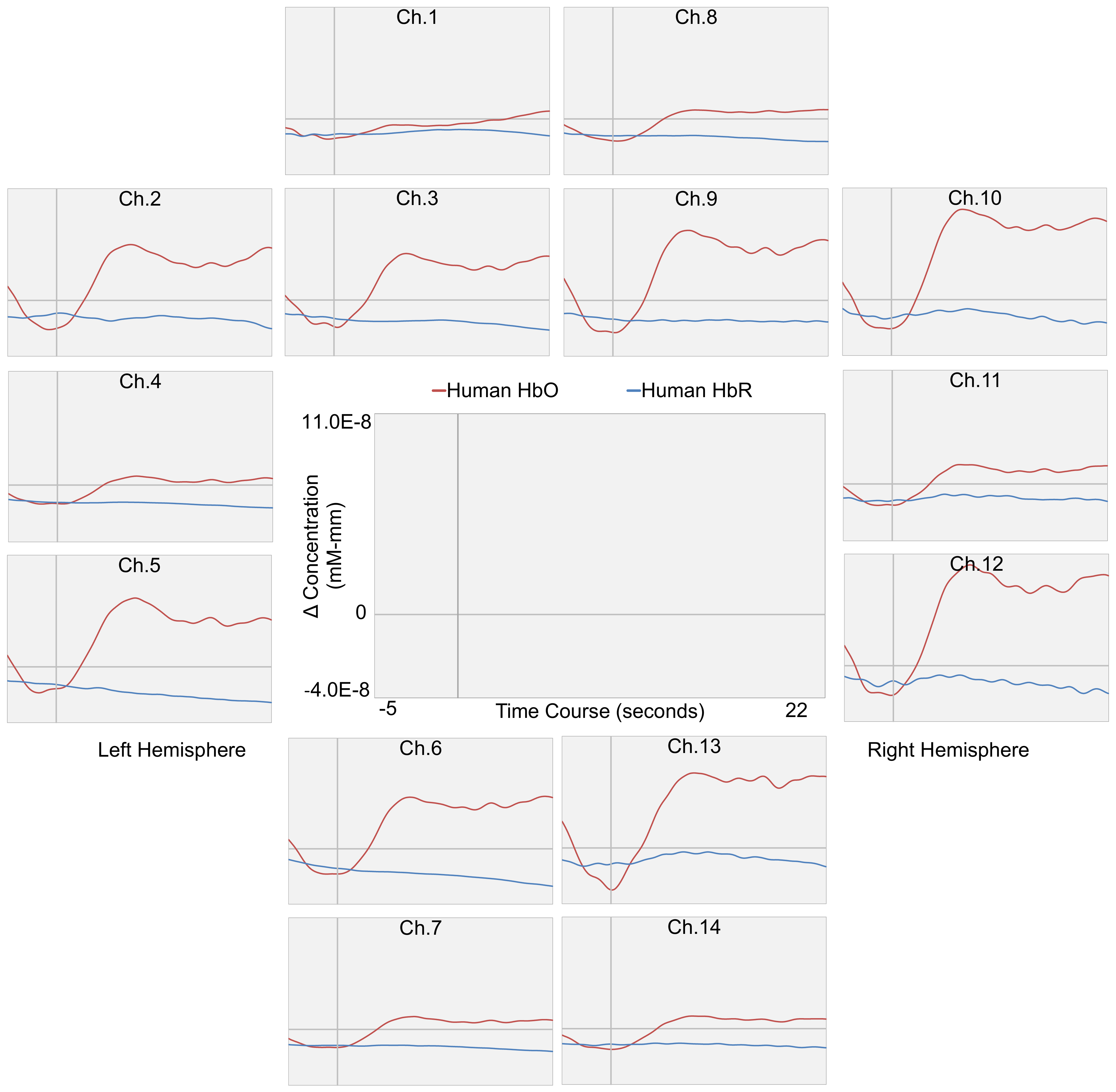

Supplement: S2 Fig — FNIRS data from sample NT participant that includes HbO and HbR time course from each channel. (TIF) [file pone.0158804.s002.tif]
