# Supplementary material for: Atypical Asymmetry for Processing Human and Robot Faces in Autism Revealed by fNIRS
Source: PLoS One. 2016 Jul 7;11(7):e0158804. doi: 10.1371/journal.pone.0158804 (PMC4936708; doi:10.1371/journal.pone.0158804)
Supplement: S1 File — (DOCX) [file pone.0158804.s003.docx]

I grant permission for use of my picture under the CC BY 4.0 license.

-Elaine Short
